# Shaping the Future Plate: Exploring the Safety Concerns of Cultivated and Conventional Meat Production

**DOI:** 10.33549/physiolres.935712

**Published:** 2026-08-01

**Authors:** Petra NEVECERALOVA, Hana RASCHMANOVA, Peter GORILAK, Jan CERNY

**Affiliations:** 1 BeneMeat Technologies, Kamenice, Czech Republic; 2 Department of Cell Biology, Faculty of Science, Charles University, Prague, Czech Republic

**Keywords:** Alternative protein, Cell cultivation, Cultivated meat, Conventional meat

## Abstract

In response to the growing global population and the unsustainable nature of current livestock agriculture, cultivated meat (CM) offers a promising alternative protein source. CM, produced through advanced tissue engineering techniques, aims to mitigate health, ethical, and environmental issues associated with conventional meat. This review evaluates the safety of CM consumption, comparing its risks and benefits to those of traditional meat. CM presents numerous advantages, including the reduction of zoonotic diseases and antibiotic resistance. However, it also introduces new challenges, such as genetic modification concerns and whether the CM can cause any kind of disease of affluence. The review highlights the necessity for rigorous safety assessments and regulatory frameworks to ensure CM’s safe integration into the food supply. Additionally, CM production’s lower environmental impact, such as low greenhouse gas emissions or reduced land and water use make it a viable solution to global food security and environmental sustainability.

## Introduction

**Fig. 1:**
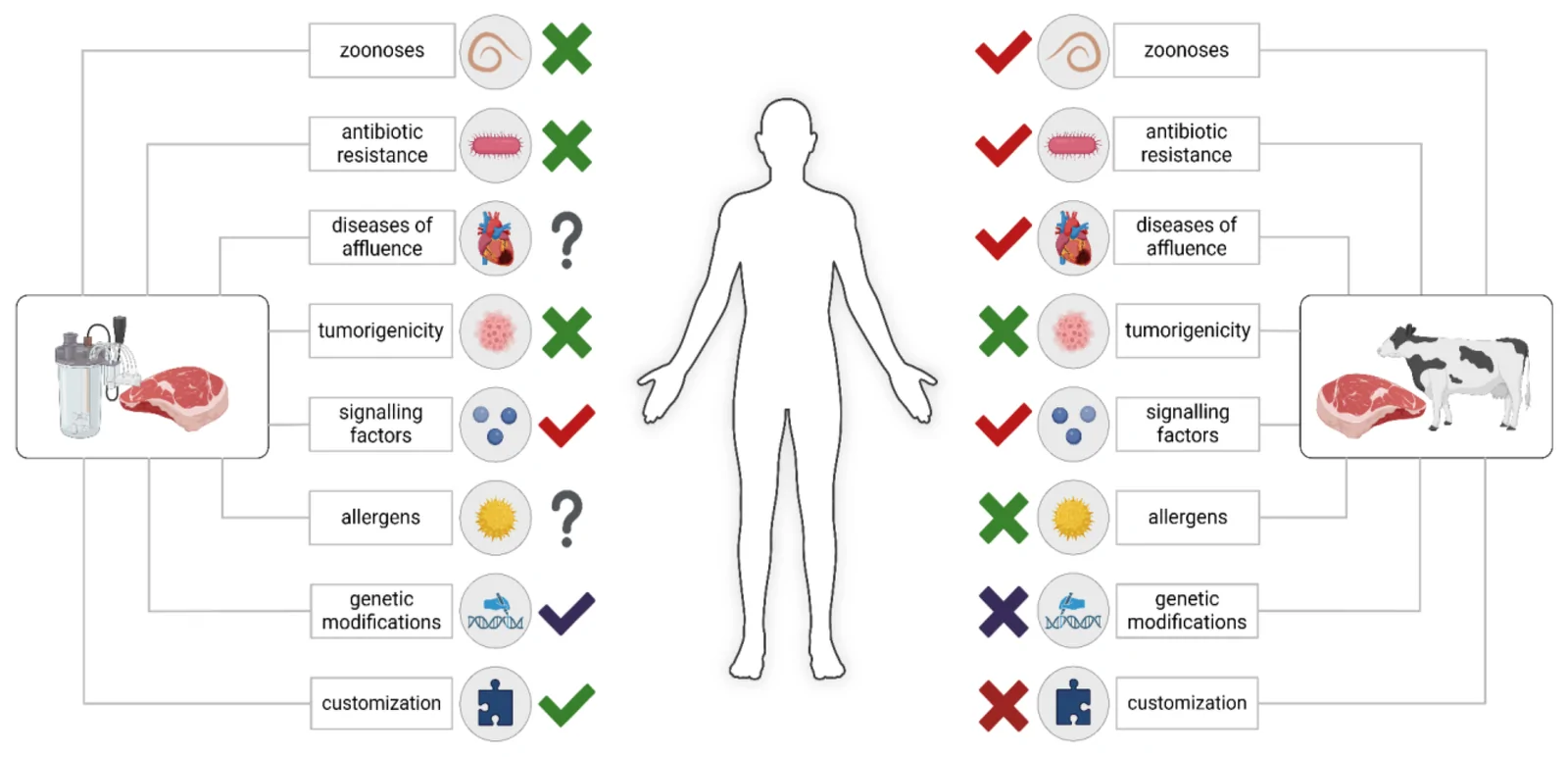
The summary of most concerned and associated risks that come with cultured meat (left panel) or conventional meat (right panel) consumption. The color of “X”, “check mark” or “question mark” symbols: green means positive outcome, red means negative, blue means neutral and grey means not validated yet. The question mark by the allergens in cultivated meat (CM) means that the presence of allergens in CM highly depends on the final product composition. Created with Biorender.com.

In the global hunt for high-quality, low-cost protein sources, given the rate of population growth, current mass livestock agriculture may no longer be sustainable. Our future depends heavily on developing new sources of food production. Exploration of new protein alternatives is necessary, and consumption of analogous or alternatively produced animal proteins has increased dramatically in recent decades [1]. A novel source of animal protein - cultivated meat - has recently entered the discussion. Initially referred to as “lab-grown” or “in-vitro meat,” terms like “cultivated meat,” “cultured meat,” and “cell-based meat” are now commonly used. For this review article, we will use “cultivated meat” (CM). Public and scientific communities alike are generally unfamiliar with CM [2], and its introduction raises numerous questions and emotions, particularly concerning the safety of CM consumption (Fig. 1). The aim of this review article is to address these questions and issues, comparing the risks and benefits of CM consumption with those of conventional meat.

## Definition of meat and cultivated meat

“Meat” typically refers to skeletal muscle of animals, a complex tissue comprising various components [3]. The European Parliament defines “meat” [4] as the edible parts of animals, including blood with no further specification. In the context of CM, categorized as novel foods, the process often involves obtaining a biopsy sample from an animal destined for human consumption. This ensures compliance with legal regulations and guarantees the safety of samples for subsequent cell isolation [1]. Cells are isolated in a sterile environment and cultivated under sterile conditions, using a medium free of animal-derived components to facilitate cell expansion. Additional steps, such as differentiation into specific cell types or genetic engineering for cell immortality, may be employed to support long-term cell proliferation. After harvesting, cells are inactivated, making them non-viable and sterilizing the final product. While CM consists of cultivated cells and may not achieve the complexity of natural muscle tissue in the near future, efforts in tissue engineering to mimic muscle architecture are ongoing, although they present significant challenges [2].

## The meat issue

Most nonhuman primates follow a plant-based diet, occasionally including invertebrates, with only chimpanzees and bonobos sometimes consuming mammalian meat [5]. Modern humans experienced a dietary shift over 2.6 million years ago, incorporating meat, which was crucial for brain development and cognitive evolution due to essential nutrients [6]. Meat provides essential nutrients, minerals, and bioactive compounds [7], yet poses health risks like cardiovascular disease [8], type 2 diabetes [9], certain cancers [10], and reduced longevity [11]. The meat industry’s practices raise ethical and environmental concerns, with over 80 billion animals slaughtered annually. Issues include mishandling, hormone use, poor housing, inadequate feeding, and ineffective stunning methods [7]. The global population is projected to reach nearly 10 billion by 2050 [12], challenging sustainable food production. Meat production, quadrupled since 1961, now stands at 340 million metric tons annually, expected to rise to 460–570 million metric tons by 2050 [13]. This demand surge, along with intensive farming and subsidies, significantly impacts the environment. From 2018 to 2020, meat production accounted for 54 % of agriculture-related greenhouse gas emissions [14] and 41 % of total agricultural water use, contributing to water shortages. Livestock farming also affects deforestation, soil degradation, and biodiversity loss [1]. These cumulative impacts necessitate reassessing meat production’s sustainability. Innovative solutions like plant-based meats and CM offer potential pathways toward a more sustainable and ethical food system.

## Health risks associated with conventional meat consumption

### Zoonoses

Meat poses a significant risk of transmitting zoonotic diseases, and 75 % of emerging human pathogens are zoonotic [15], causing over a billion human infections and economic losses of hundreds of billions annually [16].

Many bacteria in meat cause foodborne diseases. In the EU, the most common are *Campylobacter* spp. and *Salmonella* spp. [17]. Other harmful bacteria include *Listeria* spp., *Mycobacterium*, *Brucella*, and *Staphylococcus* spp., contaminating meat during processing or handling [13]. A critical concern is antibiotic resistance. Nearly all these bacteria have shown resistance to certain antibiotics [18]. Increased meat demand drives use of antibiotics in livestock for disease prevention, treatment, and growth promotion, contributing to antibiotic-resistant bacteria [19] and raising concerns about a post-antibiotic era [20].

Meat can harbor viruses like hepatitis A, norovirus, enterovirus, rotavirus, and astrovirus [21]. Hepatitis E, linked to undercooked pork and wild boar, is rising in developed countries [22]. Bovine leukemia virus, linked to human cancers, is transmitted through raw milk and undercooked beef [23]. Influenza viruses in poultry and pigs pose pandemic risks [24]. Coronaviruses from wild animals caused SARS, MERS, and COVID-19 outbreaks [25].

Endogenous viruses are embedded in the DNA of all eukaryotic genomes, usually inactivated, though some remain active [26]. They pose no zoonotic risk to humans when it comes to meat consumption [27,28]; however, further research is needed.

Prions, proteins occurring naturally in organisms, exist in non-pathogenic (PrPC) and infectious forms (PrPSc), causing diseases like bovine spongiform encephalopathy (BSE) and Creutzfeldt-Jakob disease (CJD). BSE, the only zoonotic prion disease, causes variant CJD in humans [29]. Pathogenic prions are highly resistant to sterilization [30].

Food-borne parasites pose significant risks through contaminated meat consumption [31]. In Europe, the most common are *Echinococcus multilocularis*, *Toxoplasma gondii*, *Trichinella spiralis*, *Echinococcus granulosus*, and *Cryptosporidium* spp., all zoonotic. These parasites can lead to severe health issues [32]. Prevention focuses on improved hygiene in meat production and rigorous inspection protocols.

### Diseases of affluence

In 2015, one of the world’s most prestigious medical journals, The Lancet, reported that red meat might be carcinogenic, classifying processed red meat as a group 1 carcinogen and red meat as group 2A [10]. Red meat includes unprocessed mammalian muscle, while processed meat is preserved or flavored through salting, smoking, or fermentation. Studies have linked red meat consumption to increased cancer risk [33]. High-temperature cooking forms heterocyclic aromatic amines (HAAs) [34] and polycyclic aromatic hydrocarbons (PAHs) [35], known carcinogens. Heme iron in red meat is an essential micronutrient for many cellular processes [36,37]; on the other hand, it can form N-nitroso compounds (NOCs) in the digestive tract, associated with cancer [38]. Another study suggests that naturally occurring bovine viruses in conventional meat might induce digestive system cancers, supported by data showing higher cancer rates in regions with high beef consumption, especially undercooked beef, compared to regions with lower consumption. Oncogenic viruses like polyomaviruses, papillomaviruses, and TT viruses in beef could contribute to carcinogenesis by altering signaling pathways or causing mutations [39]. Viral alterations in cells from colorectal cancer patients further support this theory [40]. These findings highlight the need for caution in red meat consumption and further investigation into carcinogenic cooking methods and meat-borne viruses.

Studies suggest a link between high meat consumption and cardiovascular diseases due to saturated fat [41], however, a meta-analysis found no significant correlation [42]. Newer research indicates that other factors, such as high salt content [43] and HAAs from cooking [44], may contribute to cardiovascular risks. Other compounds in meat processing, such as high levels of salt and phosphates, contribute to cardiovascular and kidney diseases [45]. Emerging evidence highlights the role of gut microbiota in producing trimethylamine N-oxide (TMAO) from L-carnitine in meat, linked to cardiovascular diseases and other health issues [46]. These findings suggest some molecules in meat and their interaction with gut microbiota play a significant role in health risks, but further research is needed to clarify these mechanisms.

Red meat may increase the risk of type 2 diabetes, though the mechanism is not fully understood. Heme iron in red meat induces oxidative stress and damages pancreatic beta cells, with high iron levels linked to increased diabetes risk [47]. Saturated fat content in meat has been suggested as a factor contributing to the risk of diabetes, although findings are inconclusive [48]. Excessive meat consumption can lead to weight gain and obesity, significant risk factors for type 2 diabetes [49, 50]. Advanced glycation end products (AGEs) formed during high-temperature cooking or meat processing are also implicated [51].

Nitrosamines, generated from nitrites and nitrates used as preservatives, have carcinogenic properties [52]. Dietary advanced glycosylation end-products are formed during the Maillard reaction at high temperatures [53]. Endocrine-disrupting chemicals (EDCs) from contaminated feed or plastics used in processing are another concern [54].

## Health concerns associated with CM

### Zoonoses

Overall, CM is a safer source of animal protein in the sense of reducing the risk of zoonotic diseases associated with meat consumption. The cell culture lacks an immune system, necessitating a perfectly sterile environment, with sterility requirements much higher than those in meat-processing facilities. Upscaled cultivation must also be antibiotic-free, increasing the need for stringent sterility. Due to the sterile environment, CM is less prone to microbial or viral contamination, reducing zoonotic disease risk [1].

However, contamination sources still exist, such as bacteria from donor biopsies or lapses in good laboratory practices. A significant concern is *Mycoplasma* spp., with up to 35 % of cell cultures globally affected by mycoplasma contamination, necessitating routine testing [55]. Fetal bovine serum (FBS), used in cell culture media, can carry contaminants, including mycoplasma, viral agents, and prions [56]. The use of FBS raises safety and ethical concerns, as it is sourced from bovine fetuses obtained during slaughter [57]. A shift toward synthetic, serum-free media, potentially using supplements derived from plant or microbial products, is underway [1] and is expected in commercial CM production [2].

Changes in cell line development, such as scale or media composition alterations, can activate endogenous viruses. Regular testing with qPCR techniques is recommended to ensure cultured cell safety [58]. Although endogenous bovine viruses constitute nearly 30 % of the genome [59], identifying and knocking out problematic elements is possible.

Pathogenic prions can enter cell culture via donor animals or FBS [14]. Regular prion testing or genetic modification of the PRNP gene can address this. Creating a Prnp^−/−^ cell line ensures prion-free cells. Prnp^−/−^ cell lines from mice exist, but these cells struggle with viability in serum-free media [30]. Adapting to serum-free media is crucial for CM scale-up [1]. Other methods, like RNAi techniques, can silence prion expression, but their scalability is uncertain. No Prnp^−/−^ cell lines from relevant species are currently available for CM production, according to our best knowledge.

Parasite contamination risk in CM is low, as donor animals are under veterinary supervision. Biopsy parasite detection methods reduce the likelihood of viable parasites in cell culture [1].

The safety of cultivated meat technology with regard to biological contamination is taken into account. Examples of safety practices and strategies involved in the cultivated meat technology, including occupational health practices linked to the tissue sampling [18,19,22], and the safety of work with cell lines was summarized elsewhere [60].

### Tumorigenicity and carcinogenicity

One of the concern is the potential for CM cells to migrate within the consumer’s body and form tumors, particularly since CM cells will likely be immortalized through targeted genetic modification or spontaneous processes. Immortalization typically involves upregulating telomerase (TERT) expression, which maintains telomere length and prevents cellular aging [61]. Although telomerase is naturally present in eukaryotes, including common dietary plants [62], its presence in CM is sensed as concerning. However, no chromosomal abnormalities or tumor formation were found in immunodeficient mice injected with TERT-modified cells over six months [63]. Additionally, TERT degrades during cooking and digestion, and RNA, a substrate for telomerase, breaks down in the gastrointestinal tract [64]. Another immortalization technique relies on spontaneous (random) immortalization of cells, after being exposed to the irradiation or certain chemicals [65], but this treatment often makes unwanted changes in the cells, such as chromosomal aberrations [66]. In the end, the cells used to create CM are required to be inactivated after harvesting; hence, the risk of tumorigenicity is very low, if it exists at all.

It is understandable that eating immortalized cells might raise concern. We emphasize that immortalization is not synonymous with malignancy. Many widely used lines, such as embryonic stem, HEK293, or CHO cells, achieve immortality through telomerase activity or viral mechanisms rather than malignant transformation, and have been safely used for decades in biopharmaceutical production [67,68]. Moreover, biologically, cells are not intrinsically dangerous once removed from their physiological context; standard heat treatment reduces them to denatured protein, lipids and nucleic acids. Documented cases of transmissible cancers (e.g. Tasmanian devil facial tumor disease [69] or multiple bivalve species [70]) are exceptional, occurring only in species with extreme genetic homogeneity and unique ecological conditions. In humans, rare iatrogenic tumor transmissions have occurred almost exclusively in the context of organ transplantation under strong immunosuppression. Notably, sustained xenogeneic tumor growth in experimental systems requires severe immunodeficiency [71], underscoring the improbability of such events in immunocompetent individuals.

In fact, it would be misleading to equate cultivated meat lines with “typical cancer cells.” While they share traits like deregulated cell cycle control, the evolutionary pressures of a bioreactor—favoring genomic stability and metabolic efficiency—are fundamentally different from the pressures driving in vivo tumor progression, such as immune evasion and tissue invasion [72,73]. Cell lines for cultivated meat are typically derived from primary tissue or stem populations and optimized through controlled interventions like telomerase activation [74,61]. Therefore, “modified cell line” is a biologically more accurate description of these specialized genotypes. We agree that transparency regarding immortalization strategies and rigorous tumorigenicity studies are essential to ensure absolute production safety.

### Signaling factors

Another concern is the potential presence of residual compounds with signaling or hormonal functions in the harvested and washed biomass originating from the cultivation medium. The medium typically includes signaling proteins like insulin, neuregulin 1 (NRG1), epidermal growth factor (EGF), transforming growth factor beta 1 (TGFβ1), fibroblast growth factor 2 (FGF2), insulin-like growth factor 1 (IGF1), transferrin, and albumin [75]. These proteins naturally occur in vertebrate blood and tissues, playing diverse physiological roles.

Animal-derived food products naturally contain many growth factors and hormones. For instance, milk and dairy products contain TGFβ, IGF, EGF, and FGF families and numerous hormones like insulin or estrogens [76,77]. Transferrin, albumin or NRG1 are present or produced in muscle tissue [78,79].

Residual cultivation medium ingredients that can potentially be present in the CM product must be evaluated for safety in pre-market consultations with regulatory agencies. UPSIDE Foods reported higher residual growth factor concentrations in their cultivated chicken compared to conventional poultry, but these were effectively denatured by cooking at 74 °C (165 °F). Also, the growth factors naturally occurring in the tested conventional store-bought meat sample might have already degraded during storage [64].

### Allergens

A presence of allergens might pose another safety concern of CM [80]. In CM production, allergens may come from plant or microbial-derived cultivation medium components or scaffolds. Hydrolysates or extracts obtained from plants, fungi, or microbial cells are commonly utilized in CM production as sources of macro- and micro-nutrients or signaling molecules in the cultivation medium. For instance, soybean protein hydrolysate [81] and algal hydrolysates [82] provide amino acids or glucose, while algal extracts [83] serve as supplements in serum-free cultivation media. Edible scaffolds for cell growth and differentiation can be derived from textured soy protein [84], pea protein [85], fungal mycelia [86], or algal materials [87] and others. Microalgae and fungal materials may contain allergenic components [88]. Post-harvest washing steps are crucial to reducing or eliminating compounds present in the cultivation medium [64]. Utilizing plant hydrolysates, extracts, or compounds from non-pathogenic or Generally Recognized as Safe (GRAS) microorganisms for cultivation media preparation theoretically enhances safety. However, if edible plant- or microbial-based scaffolds are retained in the final CM product, stringent safety measures will be necessary.

### Genetic modification

All food originates from plants, animals, or microbes, containing DNA, RNA, nucleotides, and free nucleic bases. We typically ingest significant amounts of DNA and RNA daily [89]. While all cells within an organism share the same DNA sequence, variations arise due to epigenetics [90]. All DNA, regardless of its origin, consists of four nucleotides and undergoes complete degradation in the digestive tract [89]. Genetically Modified Organisms (GMOs) have sparked debates primarily focused on their safety for human consumption. Concerns often revolve around health, environmental, and ethical issues [91]. Proponents argue that GMOs enhance global food security by improving crop yields, pest resistance, and nutritional content. Opponents highlight potential human health and environmental risks [92]. Rigorous safety assessments of GMOs evaluate allergenicity, toxicity, horizontal gene transfer, and nutritional value before market entry [93].

Reputable organizations like the World Health Organization and the American Medical Association have researched GMO biosafety extensively. Over 130 research projects involving 500 independent research groups over 25 years conclude that GMOs pose no greater risks than conventional breeding technologies [94]. Claims of adverse effects in animals fed with genetically modified food lack scientific support, as long-term feeding studies show GM and non-GM crops are nutritionally equivalent [95].

Two primary concerns persist about GMOs and human health. The first involves the potential for new allergens in the food supply due to allergen transfer *via* genetic modification, but rigorous testing can detect such risks early [96]. The second concern is horizontal genetic transfer—the movement of genes between different species—which could integrate foreign genes into human cells or microbiomes. So far, no studies have confirmed such transfer [97].

Genetic engineering includes transgenic and cisgenic methods. Transgenic engineering transfers genetic material between unrelated species, introducing new traits that are not achievable through conventional breeding [98]. Cisgenic methods involve editing/transferring genes within a species or its close relatives, likely posing no more risks than conventional breeding [99].

In CM production, bioengineering is crucial. Techniques like adapting cells to grow in certain conditions are used for desired traits [100]. Cisgenic genetic engineering provides precision in achieving specific cell line characteristics, for instance, knocking out genes for prion proteins or modifying the α-1,3-galacto-syltransferase gene to reduce the allergenicity of mammalian meat.

The regulatory framework for GMOs, especially in the EU, remains stringent, contrasting with approvals for foods developed by using less precise methods like spontaneous mutagenesis [101]. The pace of regulatory processes often trails behind scientific advancements, challenging effective risk evaluation and regulation [102]. With global challenges like climate change, adopting genetically modified foods could be as transformative as the rapid approval of mRNA vaccines during the COVID-19 pandemic [103].

### Nutritional value

The nutritional composition and quality of conventional meat is subject to oversight by food regulatory bodies such as the European Food Safety Authority (EFSA) in the EU and the Food Safety and Inspection Service (FSIS) in the USA. CM products must closely resemble conventional counterparts, which is pivotal in enhancing consumer acceptance of CM [104].

A comparison of cultured poultry meat (CPM) and traditional chicken cuts revealed similar amino acid levels, but differences in fatty acids, cholesterol, minerals, and vitamins. CPM produced with a serum-free medium had lower levels of 18:2w6 linoleic acid than conventional poultry, but higher concentrations of cholesterol, vitamin A, and minerals, especially phosphorus and iron. The cholesterol content in CPM was more like beef or chicken liver, while its vitamin A content is higher than conventional chicken breast but lower than chicken liver [64]. Because of potential health risks of excessive phosphorus and iron intake [105, 106], adjusting CM’s mineral content is crucial.

Microbiota digestion of L-carnitine may adversely affect the cardiovascular system [46]. Preliminary data suggest minimal expression of L-carnitine in CM-producing cells [107] as it is synthetized mainly in the liver and kidneys [108].

Since CM’s post-harvest processing and preservation methods are not fully elucidated, it is not clear whether the conventional meat preservation compounds, such as nitrites and nitrates, will be applied in the CM technology. However, CM has the potential for lower contents of EDCs compared to conventional meat, in case that pure defined nutrients are used instead of crops, which could introduce EDCs into conventional meat.

CM production uses various edible or biodegradable polymers from plants, fungi, or algae as gelling agents, fat emulsifiers, and modifiers of water binding capacity, thickness, and stability [109]. These polymers are common in the food industry, posing no significant health risks.

## Potential benefits of CM

### Customization

CM’s customization to consumer preferences is a significant advantage. CM’s conceptual framework enables the creation of innovative meat blends by combining various cell types or even cells from different species, resulting in unique properties and nutritional profiles. By cultivating cells in controlled environments with defined conditions, components linked to diseases of affluence can be reduced or eliminated. For example, CM’s fat content can be adjusted through several strategies. The proportion of cell types (e.g., myosatellite cells, adipocytes, fibroblasts) in the final product determines the fat content. Additionally, the composition of fat in cells is influenced by the cultivation medium’s composition [64]. Cellular heme-iron content, associated with health risk, can be controlled by adjusting cultivation conditions. Although CM typically contains minimal myoglobin, adding myoglobin to the cultivation medium can replicate conventional meat’s color and flavor. Alternatively, if reducing heme content is desirable for health, ambient oxygen conditions can ensure low myoglobin content, with subsequent red color addition via natural dyes like sugar beet or saffron [110]. Moreover, CM products can be enriched with essential dietary supplements like vitamin D, or compounds such as taurine, addressing common deficiencies.

### Environment crisis

**Fig. 2:**
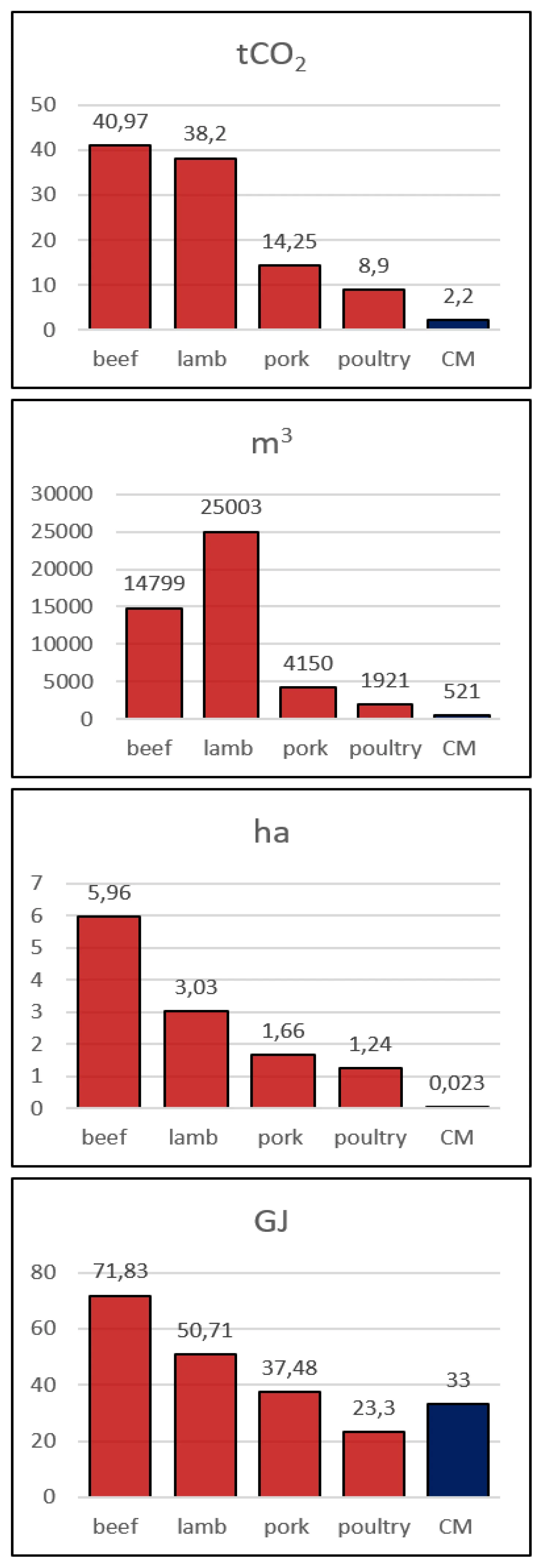
Environmental impact of different kinds of meats, including cultivated meat (CM). (a) Produced tCO_2_, (b) consumed m^3^ of water, (c) utilized ha of land, and (d) consumed energy in GJ; all the values per 1 ton of product, data for conventional meat are taken for Great Britain. According to 112.

Current agricultural practices have disrupted ecological balance, impacting ecosystem integrity, land systems, and element cycles, contributing to various ecological issues, including climate change. Traditional agriculture exacerbates environmental problems and is susceptible to climate change, while CM production offers environmental benefits. CM production requires less water and generates less pollution compared to conventional livestock farming (Fig. 2). It also necessitates significantly less land, alleviating pressures on habitat degradation [1]. One of the independently reviewed life cycle assessment (LCA) study [111] shows that land use is reduced to 3.1 m^2^/year per kilogram of meat (expected improvement to 2.0 m^2^/year) and greenhouse gas emissions are 5.28 kg of CO2 eq. per 1 kg of meat (potential to reduce to 3.29 kg CO2 eq.). The CM industry also potentially reduces the number of livestock, hence decreasing animal slaughter. Offering alternatives to bush meat, CM can address the issue of wildlife hunting, which impacts ecosystems and poses food security risks due to the heightened risk of zoonotic diseases [1].

As of 2020, an estimated 720–811 million people globally faced hunger [1]. Cellular agriculture holds promise toward a sustainable food system [2] via ensuring the access to healthy, affordable proteins, while mitigating health risks associated with meat consumption, including zoonotic diseases and antibiotic resistance [1].

## Conclusion

This review aims to address concerns associated with CM consumption and compares it with conventional meat, fostering informed discourse on this pivotal topic. It benefits regulatory bodies, the scientific community, and CM manufacturers by ensuring the safety and transparency of CM production. Additionally, it contributes to streamlining regulatory pathways that are currently under development. For consumers, CM has the potential to represent a responsible dietary choice aligning with evolving preferences. CM embodies scientific advancements, ethical considerations, and sustainable food production practices. Substituting conventional meat consumption is urgent, as traditional agriculture cannot meet the escalating demand for meat and dietary protein due to rising global population and per capita demand. However, meat remains vital to the human diet. CM is the world’s first full alternative to conventional meat.

While both CM and conventional industry aim to produce the same product: meat, CM is grown in a controlled, sterile environment, allowing greater precision and control throughout production, potentially yielding a safer product. CM represents a game-changer in animal-based food production, addressing ethical, environmental, and safety concerns. Optimized and cost-effective CM production may eliminate the need for expanding the meat industry. By using animal cells and eliminating animal-derived sera, CM prioritizes animal welfare, combats antibiotic resistance, and diminishes zoonoses. Additionally, CM lowers greenhouse gas emissions, water usage, nutrient contamination, land expansion, and other environmental challenges linked to animal-based food manufacturing.
